# The early Th17/Treg ratio predicts the immune activation set point in patients with primary HIV infection

**DOI:** 10.1186/1742-4690-9-S2-P285

**Published:** 2012-09-13

**Authors:** MF Chevalier, G Petitjean, C Didier, P Girard, L Meyer, F Barré-Sinoussi, D Scott-Algara, L Weiss

**Affiliations:** 1 Institut Pasteur; Université Paris Diderot, Paris, France; 2 Institut Pasteur, Paris, France; 3 AP-HP Hôpital Saint-Antoine, Paris, France; 4 INSERM U1018, Paris, France; 5 Université Paris Descartes; AP-HP; Institut Pasteur, Paris, France

## Background

Persistent systemic immune activation plays a central role in the pathogenesis of HIV disease. Impairment of the intestinal barrier and subsequent microbial translocation might be involved in chronic immune activation. Th17 cells are important in the maintenance of intact epithelium and host defense against extracellular pathogens. The ratio between the two closely related CD4 subsets Th17 and Tregs has been recently found to shrink with HIV/SIV disease progression. The aim of the study was to analyze, in patients with early primary HIV infection (PHI), the relationship between Th17/Treg ratio and the immune activation set point, known to predict disease progression.

## Methods

27 patients with early PHI were included in a prospective longitudinal study and followed-up for 6 months. T-cell activation and CD4^+^CD25^+^CD127^low^Foxp3^+^ Treg frequency were assessed on fresh PBMC. Th17 cells were quantified by intracellular cytokine staining on sorted peripheral CD4 T cells stimulated with PMA/ionomycin for 5h. Correlations were assessed using spearman non-parametric tests. Plasma I-FABP, a marker of mucosal damages and soluble CD14 (sCD14) were measured by ELISA.

## Results

A strong negative relationship was found at baseline between the Th17/Treg ratio and the proportion of activated CD8 T cells expressing CD38/HLA-DR (p=0.008) or Ki-67 (P=0.001). At baseline, Th17/Treg ratios also negatively correlated with sCD14 plasma levels (p=0.003). I-FABP levels, which were similar to controls at baseline, increased at month 6. The Th17/Treg ratio at baseline (but not the proportion of Th17 cells) negatively correlated with the frequency of HLA-DR^+^CD38^+^ or Ki-67^+^ CD8 T cells at month 6, defining the immune activation set point (p=0.02 and p=0.0005 respectively). sCD14 plasma levels were also found to predict the immune set point (p=0.02).

## Conclusion

Our data do not support early mucosal damages in PHI. However, the early Th17/Treg balance correlates with sCD14 levels and predicts the immune activation set point.

